# The German Communities That Care Youth Survey: dimensionality and validity of risk factors

**DOI:** 10.3389/fpubh.2024.1472347

**Published:** 2024-09-30

**Authors:** Maren Reder, Ronja A. Runge, Helge Schlüter, Renate Soellner

**Affiliations:** Institute of Psychology, University of Hildesheim, Hildesheim, Germany

**Keywords:** confirmatory factor analysis, unidimensionality, risk factor, Communities That Care, validity, criterion validity, scale evaluation, adolescence

## Abstract

**Background:**

Communities That Care (CTC) is an evidence-based community change strategy for supporting healthy youth development. One of its key elements is the development of a community profile to identify and prioritise risk factors for health and behavioural problems in adolescents based on the CTC Youth Survey. The strategy was originally developed and evaluated in the United States. An adapted version of the survey has been used in Germany since its first implementation in 2008. However, the dimensionality and validity of the adapted risk factor scales have not yet been evaluated. Therefore, this study aimed to confirm the assumed unidimensional structure and to establish the concurrent criterion validity of each risk factor.

**Methods:**

A sample of 1,911 adolescents attending grades six to eleven in Lower-Saxony, Germany, was used to evaluate 23 risk factor scales of the German CTC Youth Survey covering the domains peer/individual, family, school, and community. Confirmatory factor analysis was used to assess the dimensional structure of all risk factors with sufficient item numbers. Goodness of fit was determined using CFI, SRMR, and RMSEA. Latent regression analysis tested the concurrent criterion validity of all unidimensional risk factors. For this, violent and delinquent behaviour, substance use, and depressive symptomatology were regressed on each risk factor.

**Results:**

All evaluable risk factors demonstrated acceptable to good model fit regarding unidimensionality and predicted violent and delinquent behaviour, and substance use better than depressive symptomatology. Regarding the peer/individual risk factors, there are particularly high correlations with violent and delinquent behaviour, and substance use. In contrast, two risk factors were not correlated with substance use, whilst two other risk factors were not correlated with depressive symptomatology.

**Conclusion:**

Overall, the results indicate that most risk factors demonstrate unidimensionality and are valid in terms of concurrent criterion validity. Although some risk factors were not correlated with every outcome, they still predicted most outcomes, suggesting that the CTC Youth Survey is a viable tool for communities to assess their community risk profile. Risk factors that did not demonstrate unidimensionality or concurrent criterion validity should be monitored in future surveys and interpreted with caution until further evidence is available.

## 1 Introduction

Community-based prevention is an important strategy for supporting and facilitating healthy youth development. There are numerous preventive interventions available for narrowly defined outcomes, often with a clinical focus, rather than promoting healthy development in general. For instance, reviews on community-based prevention, aimed at adolescents, have been published on topics such as dental health (1), obesity (2), sexual violence (3), depression and anxiety (4), and self-harm and suicide (5). Whilst some preventive interventions focus on a single outcome, others yield multiple outcomes. Communities face the highly challenging task of deciding which of the various prevention interventions best fit their needs. Therefore, community prevention systems that assist communities in selecting the most appropriate prevention interventions, following a needs assessment, as well as coordinating prevention efforts, are vital. One community prevention system that is comprehensive (6), evidence-based (7), and cost-effective (8) is Communities That Care (CTC).

CTC was developed in the United States in the 1980s by the Social Development Research Group (9, 10). It assumes that several risk and protective factors influence the occurrence of potentially health-compromising problem behaviours. The risk and protective factors, taken into account in the CTC framework, were identified by reviewing studies on adolescent drug use, delinquency, and violence (11). The aims of CTC are to reduce risk factors and enhance protective factors in order to prevent adolescent problem behaviour (9, 12), and to create good conditions for a safe and healthy childhood (13) by supporting the communities to select appropriate evidence-based programmes.

The CTC Youth Survey is a crucial component of CTC. It is used to measure risk and protective factors and to create risk and protective factor profiles for communities, enabling them to select prevention interventions that are tailored to their specific needs. Beyond that, this survey also measures problem behaviours (14). The CTC Youth Survey is theoretically based on the Social Development Model, which is grounded in criminological theory (15). Within this model, hypotheses regarding problem behaviour are formulated on the basis of research results on risk and protective factors. Notably, the “problem behaviours” of the CTC Youth Survey cannot always be considered “behaviours,” (e.g., depressive symptomatology) (16). Therefore, we will refer to them as “problem areas” in the following.

The CTC Youth Survey was designed to be used by 6th to 11th-grade students in the United States. The original survey underwent rigorous development steps, including the formation of an item pool, cognitive pretesting, pilot testing in classrooms, selection of items and scales using data from a probability sample of children from public schools, and assessment of the reliability and validity of these risk and protective factors (11). The risk and protective factors that had been shown in previous research to be related to drug use, violence, and delinquency, in at least two longitudinal studies, covered constructs from four domains: peer/individual, family, school, and community. The hypothesised mechanism by which risk and protective factors affect outcomes is different. Protective factors do not only affect outcomes directly, but also may buffer detrimental effects on children (17). Since risk factors are more prominent in public health discussion, in this paper we will focus on the validity of all risk factors (whilst exploring protective and/or buffering effects of protective factors will be addressed in separate research).

The original U.S. survey instrument was revised to include 20 risk factors after qualitative and quantitative data reduction procedures. Further analysis confirmed the dimensionality and validity of all 20 risk factors in the original U.S. CTC Youth Survey (11). The dimensionality of the risk factors was assessed using a two-phase Principal Component Analysis (11). Validity was assessed by examining the relationships between each of the scales measuring risk and protective factors and problem behaviour outcomes. Five of the identified factors, namely Laws and Norms Favourable to Drug Use, Transitions and Mobility, Poor Family Management, Family Attachment, and Early Initiation of Antisocial Behaviour, did not meet the criterion of unidimensionality for some grade level by gender combinations. Overall, the authors concluded to retain each of these scales as a single scale. Correlations with problem areas turned out as hypothesised and highest with scales in the Peer-Individual domain (11). To date, to our knowledge, scales regarding problem behaviour were not tested regarding unidimensionality [except for depressive symptomatology which confirmed a single factor (18)].

In Germany, CTC was adapted, in 2008, as part of a pilot project (Social Prevention In Networks, SPIN) and first implemented and tested at three sites in Lower Saxony (19). The German version of the CTC Youth Survey was based on the 2006 U.S. version and the 2004 Dutch Survey (19). A pre-test was performed with two school classes in 2009 (19). An evaluation of the pilot project indicated that CTC was implementable in German communities (19). The questionnaire used in the CTC pilot project underwent further revisions and was subsequently administered in a state-wide representative survey in Lower Saxony in 2019.

To evaluate a scale, testing dimensionality and validity is essential (20). Different methods can be used to test this (21). For the CTC Youth Survey, the most appropriate method for assessing dimensionality is confirmatory factor analysis, and for assessing concurrent criterion validity, latent regression analysis. Unidimensionality is an important prerequisite for interpreting test results (22). If items are summed or aggregated into a mean score, each item should be a good indicator of the construct. Criterion validity of risk factors is crucial, particularly if decisions are based on survey results.

Although established for the U.S. survey, the dimensionality and validity of the scales in the German version of the CTC Youth Survey must be examined due to the cultural differences between the United States and Germany. For example, societal tolerance of underage drinking and early sexual activity differs between the United States and Europe (6), as do attitudes towards the use of substances such as tobacco or illegal drugs, and dropping out of school (6). Furthermore, it is possible that the translation of the U.S. items may have affected the validity of the included risk factors.

To date, only two studies, focusing on the family domain, have been published regarding the dimensionality or validity of the scales in the German version of the CTC Youth Survey. One of these studies evaluated the psychometric properties of the risk and protective factor scales in adolescents who were hospitalised following acute alcohol intoxication (23). Of the seven family domain factors, three demonstrated good psychometric properties as unidimensional scales. One factor, family attachment, showed a factor structure with two other factors: attachment to mother and attachment to father. The second study tested the family risk factor ‘family attachment’ with data from the representative CTC Youth Survey in Lower Saxony (24). The same two-factor solution, representing attachment to the mother and attachment to the father, emerged.

The CTC Youth Survey was adapted to the Dutch context in the Netherlands (25), and the first survey was conducted in 2001. Due to geographical proximity and cultural overlap, data from the Dutch version could be considered more comparable to the German version than other language versions. However, none of the publications on CTC in the Netherlands (25–27) cover validity. Thus, despite its regular use in Germany, most risk factors of the German version of the CTC Youth Survey have not yet been tested for their validity, leaving an important research gap.

To ensure the current and future use of the German version of the CTC Youth Survey, it is essential to evaluate dimensionality and validity of all risk factors within CTC practise (i.e., all risk factors equally used as potential predictors for all problem areas) in the general population for which the survey is intended. Therefore, this study aimed to achieve two objectives: (1) to confirm the assumed unidimensional structure of the risk factors and problem areas through confirmatory factor analysis and (2) to establish the concurrent criterion validity of each risk factor with four problem areas through latent regression analysis.

## 2 Materials and methods

### 2.1 Sampling and participants

To obtain a representative sample of 7,000 students in grades six to eleven in Lower Saxony, we used a stratified random process to select classes. The sample was designed to be representative across grades, school types, and population size of the community. Information on students, classes, and schools in the 2018/2019 school year was provided by the State Education Authority of Lower Saxony. Data on population size was obtained from the Federal Statistical Office of Germany. In 2019, in Lower Saxony, 426,168 students attended public and private schools in grades six to eleven, excluding vocational and special schools. The CTC Youth Survey invited a sample of 7,000 students selected through the above described stratified random process, of which 2,191 participated. Data of 280 students had to be excluded due to missing or implausible demographic information, large proportion of missing data, short time spent on the questionnaire and/or reporting dishonest responses. The final sample consisted of 1,911 students, of whom 45.3% were boys, 54.0% were girls, and 0.8% were other. The mean age was 14.2 years, and 93.4% were born in Germany, 0.3% in Turkey, 0.7% in Russia, 0.9% in Poland, and 4.6% in other countries. 19.8% of the students attended grade 6, 15.5% attended grade 7, 24.0% attended grade 8, 13.9% attended grade 9, 16.5% attended grade 10, and 10.3% attended grade 11. The mean self-rated socio-economic status was 6.4 (SD = 1.4) on a scale of 1 to 10, with larger values indicating higher socio-economic status.

### 2.2 Measures

Twenty-three risk factors covering the domains of peer/individual, family, school, and community and four problem areas from the 2019 CTC Youth Survey were analysed in this study (please refer to Supplementary File S1 for a list of items). Table 1 describes the risk factors for all domains. With 10 factors the individual/peer domain is the most important domain (rebelliousness, early initiation of antisocial behaviour, early initiation of drug use, attitudes favourable to drug use, attitudes favourable to antisocial behaviour, peer drug use, peer antisocial behaviour, peer rewards for antisocial behaviour, sensation seeking, perceived risks of drug use), six community domain risk factors (low neighbourhood attachment, community disorganisation, transitions and mobility, perceived availability of drugs, perceived availability of handguns, laws and norms favourable to antisocial behaviour), followed by five family domain risk factors (family history of antisocial behaviour, poor family management, family conflict, parental attitudes favourable to drug use, parental attitudes favourable to antisocial behaviour), and two school domain risk factors (academic failure, low commitment to school). Usual response options were NO!, no, yes, YES! and scaling provides no categorisation nor recoding unless indicated otherwise.

**Table 1:** Risk factor scales.

Risk factor^a^	*n* items	Description and deviating response options/scaling^b^
IR1—Rebelliousness	3	Not sticking to societal rules and testing boundaries
IR3—Early initiation of antisocial behaviour	14	Respondents’ age when antisocial behaviour was shown for the first time; categorised and recoded so that young age indicates high risk
IR4—Early initiation of drug use	8	Respondents’ age when substance use was shown for the first time; categorised and recoded so that young age indicates high risk
IR5—Attitudes favourable to drug use	5	Perceiving substance use in others of the same age to be right; response options “totally wrong—wrong—right—totally right”
IR6—Attitudes favourable to antisocial behaviour	5	Perceiving antisocial behaviours in others of the same age to be right; response options “totally wrong—wrong—right—totally right”
IR7—Peer drug use	4	Having peers who engage in substance abuse
IR8—Peer antisocial behaviour	8	Having peers who engage in delinquent or violent behaviour
IR9—Peer rewards for antisocial behaviour	4	Concerns peer behaviour and social recognition
IR10—Sensation seeking	3	Enjoying risky and thrilling behaviours
IR12—Perceived risks of drug use	4	Perceiving substance use as a low risk behaviour; response options “no risk – low risk – increased risk – high risk”; items were recoded so that low perceived risk from drug use indicates a high risk factor
FR1—Family history of antisocial behaviour	4	Having family members showing different problem behaviours; response options “no –yes”
FR2—Poor family management	8	Parents’ inability to provide adequate supervision and to properly direct behaviour; items recoded so that low family management indicates a high risk factor
FR3—Family conflict	3	Having a family showing high conflict behaviour
FR4—Parental attitudes favourable to drug use	3	Drinking alcohol, smoking cigarettes and using hash/marijuana; response options “totally wrong—wrong—a little bit wrong—not wrong at all”
FR5—Parental attitudes favourable to antisocial behaviour	4	Skipping school, stealing, breaking things, and fighting; response options “totally wrong—wrong—a little bit wrong—not wrong at all”
SR1—Academic failure	2	Overall school performance in the last year “overall very good—overall good—overall satisfactory—overall sufficient—overall unsatisfactory—overall insufficient”; one item on doing worse than ones classmates with the usual response options
SR2—Low commitment to school	7	Disliking school and perceiving schoolwork as irrelevant; response options “never—rarely—now and then—often—always”; some items recoded as positive and negative attitudes towards school were assessed; one item assessed the number of days school was skipped and was categorised
CR1—Low neighbourhood attachment	3	Low levels of bonding to the neighbourhood; some items recoded as positive and negative attitudes towards the neighbourhood were assessed
CR2—Community disorganisation	6	Neighbourhood has physical deterioration and high rates of adult crime; some items were recoded as positive and negative attitudes towards the neighbourhood were assessed
CR3—Transitions and mobility	4	2 items on having changed home or school in the past year with the response options “no –yes”; 2 items on the number of moves and schools visited where response is a number
CR4—Perceived availability of drugs	5	Availability of different legal and illegal substances; response options “very difficult—difficult—easy—very easy”
CR5—Perceived availability of handguns	1	Perceived ease to get a handgun; response options “very difficult—difficult—easy—very easy”
CR6—Laws and norms favourable to antisocial behaviour	3	Normative attitudes about reacting to antisocial behaviour

^a IR2 and IR11 were not part of this CTC Youth Survey. b Usual response options “NO! — no — yes — YES!”; usual scaling involves no categorisation or recoding.^

The problem areas include violence, delinquency, substance use, and depressive symptomatology. Violence was assessed with six items responding to the questions “Have you done the following things in the past 12 months?”: “intentionally broken something that does not belong to you?,” “joined a violent or criminal youth gang?,” “been involved in a fight?,” “attacked someone to seriously hurt him or her?,” “threatened someone to get money?,” “had a weapon with you (e.g., a knife)?.” Response options were yes/no.

Delinquency was assessed with seven items responding to the questions “Have you done the following things in the past 12 months?”: “stolen something from a store?,” “been arrested by the police?,” “sold stolen items?,” “stolen something at school?,” “sprayed graffiti on someone else’s property?,” “illegally downloaded music or movies from the internet?,” “bullied someone at school or online?.” Response options were yes/no.

Substance use was assessed with eight items responding to the questions “Have you ever tried such things yourself? If so, how often in the last 4 weeks?”: “beer,” “wine / sparkling wine,” “mixed drinks,” “hard liquor,” “cigarettes/tobacco (shisha, pipe, snus),” “hash/marijuana,” “other illegal drugs (ecstasy, speed, LSD, cocaine, crystal, or heroin),” “prescription drugs without a prescription from a doctor (e.g., tranquillisers, stimulants, or painkillers).” Response options were never, 0 times in the last 4 weeks, 1–2 times in the last 4 weeks, 3–5 times in the last 4 weeks, 6–9 times in the last 4 weeks, 10–19 times in the last 4 weeks, 20 times or more in the last 4 weeks. Additionally, the item “Please think back to the last 4 weeks. During this time, how often have you had 5 or more alcoholic drinks in one evening?” was used. Response options were: not at all, 1 to 2 times, 3 to 5 times, 6 to 9 times, 10 to 19 times, 20 times or more.

Depressive symptomatology was assessed with the items “Sometimes I think my life is worth nothing.”, “Sometimes I think I’m good for nothing.”, “I often think I’m a failure”, “For the past year, I’ve felt depressed or sad most days, although some days I’ve felt okay.” Response options were: NO!, no, yes, YES!.

### 2.3 Data collection

The 2019 CTC Youth Survey received approval from the State Education Authority of Lower Saxony and the Ethics Committee of the University of Hildesheim. Study information and parental consent letters were sent to the schools of the sampled classes. If the respective head teachers and class teachers agreed to participate, they would inform parents by handing out information and consent sheets about the study and ask for their written consent for their child’s participation. The signed declarations of consent from the parents were kept at the respective school. The CTC Youth Survey was conducted online and completed by students during a single school lesson. Non-participation did not have any negative consequences for the students. Students were permitted to stop the survey at any time and skip questions.

The 2019 CTC Youth Survey was programmed and hosted by the German Research Centre for Artificial Intelligence. Each teacher was provided with a list of 35 single use one-time passwords (OTPs) for the online questionnaire. Each OTP allowed for one response. The first page of the online questionnaire provided information about the study and asked for the students’ consent to participate (tick a box). On the second page, the students were given instructions on how to complete the questionnaire.

### 2.4 Statistical analysis

The ‘lavaan’ package version 0.6–11 (28) was used in R Studio version 1.1.463 for the analyses. The analysis consisted of two steps: Confirmatory Factor Analysis (CFA) and latent regression analysis. Residual correlations between the items were modelled based on *a priori* assumptions for all models. The analysis was performed separately for each risk factor since the CTC Youth survey was designed to assess separate risk factors, not risk factor domains.

Initially, we tested unidimensionality for scales with more than three items (risk factor and problem area scales). Four independent raters (RR, HS, MR, NF) evaluated *a priori* which residual correlations were to be expected based on similarity of item content (e.g., “drunk beer” and “drunk wine/champagne”). Residual correlations that were expected by at least two of the four raters were included and estimated in the CFA models (i.e., unequal from zero). The fit of a one-factor solution was evaluated for each scale.

We used the Weighted Least Square Mean and Variance Adjusted Estimator (WLSMV) to estimate model parameters, as recommended for ordinal data with medium sample sizes (29). Scale setting was achieved using the fixed factor method. The model fit was evaluated using the likelihood ratio test statistic (χ^2^), the Comparative Fit Index (CFI), the Root Mean Square Error of Approximation (RMSEA), and the Standardised Root Mean Residual (SRMR). To interpret our results, we refer to the most conservative values: CFI: *> 0.*95 (30, 31); RMSEA: < 0.06 (30) with CI lower bound ≤ 0.05 and upper bound ≤ 0.10 though according to the authors model specifications, degrees of freedom, and sample size influence the usefulness of a set cut-off (32); SRMR: < 0.08 (30). Model fit was interpreted as overall acceptable, if 2 out of these 3 indicated acceptable model fit. Pairwise deletion was used for missing data. The robust model fit indices of ‘lavaan’ are reported.

In the second step, we conducted latent regression analysis to model a direct path in SEM between the latent risk factor and the latent outcomes. If the CFAs yielded satisfactory model fits, the respective risk factors were included in the analyses of concurrent criterion validity. Based on CTC practise, for each risk factor, we modelled relationships with all problem areas (i.e., delinquency, violence, depressive symptomatology, and substance use) in one model. To evaluate the concurrent criterion validity of the CTC risk factors, we used model fit as well as the significance and size of standardised *β* of regression paths.

At this point, it is important to note that scales with less than four items, such as IR1, IR10, FR3, FR4, SR1, CR1, CR5, and CR6, could not be tested for unidimensionality. However, we conducted an exploratory test of their concurrent criterion validity as described above. In order to calculate the concurrent criterion validity of the one-item risk factor perceived availability of handguns (CR5), we dichotomised the item into low (very difficult, difficult) and high (easy, very easy).

## 3 Results

### 3.1 Dimensionality

The CFI shows that all 15 evaluable risk factors and four problem areas achieved good model fit (please refer to Table 2 for the model fit and Supplementary File S1 for item wording and λ). However, for six scales, one of the three model fit indices indicated unacceptable fit. Specifically, attitudes favourable to drug use (IR5), attitudes favourable to antisocial behaviour (IR6), low commitment to school (SR2), and community disorganisation (CR2) showed unacceptable model fit with an RMSEA of 0.8–0.9. Additionally, the model fit for early initiation of antisocial behaviour (IR3) was deemed unacceptable based on the SRMR. Similarly, early initiation of drug use (IR4) only achieved good model fit after excluding two items (f37.8.2, other illegal drugs; f37.10.2, prescription drugs without a prescription). However, all other nine scales demonstrated good model fit according to all model fit indices. Regarding local model fit, all items had standardised loadings (λ) greater than 0.4, indicating adequate (33) item discrimination (see Supplementary File S1).

**Table 2:** Unidimensionality of risk factors and problem areas.

Scale	*n* items	*n* residual correlations	*χ*2	*df*	*p*	CFI	RMSEA [90% CI]	SRMR
IR3—Early initiation of antisocial behaviour	14	3	122.465	74	<0.001	0.981	0.019 [0.012, 0.024]	0.180
IR4^a^—Early initiation of drug use	6	2	25.055	7	0.001	0.998	0.038 [0.023, 0.055]	0.024
IR5—Attitudes favourable to drug use	5	1	62.023	4	<0.001	0.991	0.087 [0.069, 0.107]	0.039
IR6—Attitudes favourable to antisocial behaviour	5	1	44.971	4	<0.001	0.992	0.073 [0.055, 0.093]	0.031
IR7—Peer drug use	4	0	3.933	2	0.001	0.998	0.056 [0.031, 0.085]	0.044
IR8—Peer antisocial behaviour	8	5	41.590	15	<0.001	0.994	0.030 [0.020, 0.042]	0.034
IR9—Peer rewards for antisocial behaviour	4	1	4.109	1	0.043	1.000	0.041 [0.006, 0.084]	0.041
IR12—Perceived risks of drug use	4	1	0.354	1	0.552	1.000	0.000 [0.000, 0.052]	0.003
FR1—Family history of antisocial behaviour	4	1	0.401	1	0.527	1.000	0.000 [0.000, 0.053]	0.007
FR2—Poor family management	8	6	67.723	14	<0.001	0.990	0.046 [0.035, 0.057]	0.024
FR5—Parental attitudes favourable to antisocial behaviour	4	0	10.851	2	0.004	0.992	0.049 [0.023, 0.080]	0.026
SR2—Low commitment to school	8	4	264.178	16	<0.001	0.979	0.090 [0.081, 0.100]	0.047
CR2—Community disorganisation	6	3	60.081	6	<0.001	0.993	0.071 [0.055, 0.088]	0.027
CR3—Transitions and mobility	4	1	5.881	1	0.015	0.991	0.051 [0.018, 0.093]	0.034
CR4—Perceived availability of drugs	5	3	1.662	2	0.436	1.000	0.000 [0.000, 0.044]	0.003
Violence	6	2	4.682	7	0.699	1.000	0.000 [0.000, 0.022]	0.026
Delinquency	7	3	12.500	11	0.327	0.999	0.008 [0.000, 0.026]	0.039
Substance use	9	10	101.811	17	<0.001	0.998	0.052 [0.043, 0.062]	0.065
Depressive symptomatology	4	1	0.009	1	0.924	1.000	0.000 [0.000, 0.022]	0.000

a Items f37.8.2 and f37.10.2 were excluded.

### 3.2 Validity

Table 3 presents the concurrent criterion validity of all unidimensional risk factors. Table 4 displays the exploratory analysis of concurrent criterion validity of risk factors with three or fewer items, for which dimensionality could not be assessed. Only moderate to strong correlations (*β* > 0.3) are bolded. All model fits for the concurrent criterion validity calculations are contained in Supplementary File S2.

**Table 3:** Concurrent criterion validity of unidimensional risk factors.

Scale	Violence		Delinquency		Substance use		Depressive symptomatology	
	*β*	*p*	*β*	*p*	*β*	*p*	*β*	*p*
IR3^a^—Early initiation of antisocial behaviour	–	–	–	–	**0.614**	<0.001	0.236	<0.001
IR4^b^—Early initiation of drug use	**0.407**	<0.001	**0.615**	<0.001	–	–	0.118	<0.001
IR5—Attitudes favourable to drug use	**0.474**	<0.001	**0.653**	<0.001	0.**961**	<0.001	0.156	<0.001
IR6—Attitudes favourable to antisocial behaviour	**0.666**	<0.001	**0.619**	<0.001	**0.422**	<0.001	0.206	<0.001
IR7—Peer drug use	**0.540**	<0.001	**0.639**	<0.001	**0.957**	<0.001	0.102	<0.001
IR8—Peer antisocial behaviour	**0.818**	<0.001	**0.743**	<0.001	**0.694**	<0.001	0.149	<0.001
IR9—Peer rewards for antisocial behaviour	**0.470**	<0.001	**0.501**	<0.001	**0.723**	<0.001	0.220	<0.001
IR12—Perceived risks of drug use	**0.359**	<0.001	**0.438**	<0.001	**0.582**	<0.001	0.050	0.069
FR1—Family history of antisocial behaviour	**0.336**	<0.001	**0.503**	<0.001	0.**569**	<0.001	0.203	<0.001
FR2—Poor family management	**0.347**	<0.001	**0.468**	<0.001	**0.624**	<0.001	0.199	<0.001
FR5—Parental attitudes favourable to antisocial behaviour	**0.512**	<0.001	**0.480**	<0.001	**0.309**	<0.001	0.232	<0.001
SR2—Low commitment to school	**0.456**	<0.001	**0.574**	<0.001	**0.523**	<0.001	0.247	<0.001
CR2—Community disorganisation	**0.426**	<0.001	**0.395**	<0.001	**0.337**	<0.001	0.298	<0.001
CR3—Transitions and mobility	0.225	<0.001	0.213	<0.001	0.059	0.193	0.153	<0.001
CR4—Perceived availability of drugs	**0.487**	<0.001	**0.570**	<0.001	**0.747**	<0.001	0.178	<0.001

*β > 0.3 is bolded.*

a Model calculation was only possible for substance use and depressive symptomatology due to nonconvergence.

b Model calculation was only possible for violence, delinquency, and depressive symptomatology due to non-convergence.

**Table 4:** Concurrent criterion validity of risk factors with 3 or less items.

Scale	Violence		Delinquency		Substance use		Depressive symptomatology	
	*β*	*p*	*β*	*p*	*β*	*p*	*β*	*p*
IR1—Rebelliousness	**0.609**	<0.001	**0.687**	<0.001	**0.547**	<0.001	**0.334**	<0.001
IR10—Sensation seeking	**0.617**	<0.001	**0.677**	<0.001	**0.668**	<0.001	0.183	<0.001
FR3—Family conflict	0.286	<0.001	**0.312**	<0.001	0.187	<0.001	**0.492**	<0.001
FR4—Parental attitudes favourable to drug use	0.261	<0.001	**0.397**	<0.001	**0.856**	<0.001	0.084	0.006
SR1—Academic failure	0.280	<0.001	**0.305**	<0.001	0.122	0.001	**0.301**	<0.001
CR1—Low neighbourhood attachment	0.159	<0.001	0.197	<0.001	0.097	0.005	**0.352**	<0.001
CR5^a^—Perceived availability of handguns	**0.874**	<0.001	**0.833**	<0.001	**0.728**	<0.001	0.096	0.326
CR6—Laws and norms favourable to antisocial behaviour	0.196	<0.001	0.157	<0.001	0.020	0.584	0.129	<0.001

*β > 0.3 is bolded.*

^aCR5 comprises only one item; this was dichotomised as low/high to allow calculation of concurrent criterion validity. Due to no or very few cases in the substance use categories indicating frequent use, model calculation was only possible when those substance use categories were collapsed to > 6 time in the last 4 weeks.^

For certain combinations of risk factors and problem areas, the model did not converge due to their correlation being at the border of the parameter space (i.e., approaching 1). This occurred with early initiation of antisocial behaviour (IR3) in relation to violence and delinquency, as well as with early initiation of drug use (IR4) in relation to substance use.

All peer/individual risk factors for which calculation was possible showed moderate to strong associations with violence, delinquency, and substance use. For depressive symptomatology, this was only the case for rebelliousness (IR1). Perceived risks of drug use (IR12, *β* = 0.050, *p* = 0.069) was the only individual risk factor that did not show a significant correlation with depressive symptomatology.

Out of the five family risk factors, family history of antisocial behaviour (FR1), poor family management (FR2), and parental attitudes favourable to antisocial behaviour (FR5) demonstrated moderate to strong correlations with violence and substance use. Additionally, substance use was correlated with parental attitudes favourable to drug use (FR4). All five family risk factors showed moderate to strong correlations with delinquency, but only family conflict (FR3) was correlated with depressive symptomatology.

Amongst the two school risk factors, academic failure (SR1) was found to be correlated with delinquency and depressive symptomatology, whilst low commitment to school (SR2) showed moderate correlations with violence, delinquency, and substance use.

Out of the six community risk factors, community disorganisation (CR2), perceived availability of drugs (CR4), and perceived availability of handguns (CR5) showed moderate to strong correlations with violence, delinquency, and substance use. Depressive symptomatology was only moderately correlated with one risk factor (low neighbourhood attachment, CR1). Perceived availability of handguns (CR5) did not show a significant correlation (*β* = 0.069, *p* = 0.326) with depressive symptomatology. There were no significant associations of at least moderate level between transitions and mobility (CR3) and laws and norms favourable to antisocial behaviour (CR6). Furthermore, these two risk factors (CR3, *β* = 0.059, *p* = 0.193; CR6, *β* = 0.020, *p* = 0.584) did not exhibit a significant correlation with substance use.

In summary, violence, delinquency and substance use are more predictable through the risk factors than depressive symptomatology; correlations between the risk factors and depressive symptomatology are weaker. The risk factors for family, school, and community vary between low and moderate to high correlations, whilst for the peer/individual risk factors, all correlations with violence, delinquency, and substance use are moderate to high.

## 4 Discussion

### 4.1 Dimensionality

In this initial evaluation of the hypothesised unidimensionality of all risk factors in the German CTC Youth Survey, most scales fit well according to all assessed model fit indices. For some scales, only two out of three model fit indices indicated sufficient model fit. Early initiation of antisocial behaviour (IR3) has very low *n* on most items, meaning that all ordinal categories except ‘no onset’ have very few cases. Therefore, it is questionable whether these items should be dichotomised (no onset / onset) or not assessed at all due to the low n for identifying (sub-)populations at risk.

Attitudes favourable to drug use (IR5) has been modified since the 2019 CTC Youth Survey removing the Item “favourable attitude towards using prescription drugs without prescription” as this substance was of low prevalence in the surveyed age groups and not part of the original CTC Youth Survey. This may improve IR5 model fit in analyses of subsequent survey data. Attitudes favourable to antisocial behaviour (IR6) may be problematic for building a mean scale score as it combines favourable attitudes towards skipping school (Item f19.9 “skips school” shows the lowest loading of this risk factor) with favourable attitudes towards violence and delinquency.

After the 2019 CTC Youth Survey, low commitment to school (SR2) has been modified by removing the item “number of days school was skipped.” This change was made because this item overlaps with the problem behaviour of “missing school,” and using it to build the risk factor and problem area simultaneously is not recommended. This modification may improve the SR2 model fit in analyses of subsequent survey data.

Community disorganisation (CR2) might be problematic as it measures specific problems in the neighbourhood (e.g., graffiti, fights) but also contains one item on general feeling of safety (“I feel safe in my neighbourhood”). This item shows the lowest loading. Excluding or isolating this item as a separate risk factor might be advisable for future surveys.

Additionally, modifications were made to one scale (IR4). Specifically, items f37.8.2 (other illegal drugs) and f37.10.2 (prescription drugs without a prescription) were excluded from the scale due to the low number of students reporting early onset of these substances. It is important to note that this scale was not included in either the original CTC Survey (11) or the current U.S. Survey (16).

Overall, we accepted all factors as hypothesised to be unidimensional because the pattern of model fit (i.e., at least 2 out of 3 fit indices) and local fit was overall favourable. When comparing our results to previous research on the CTC Youth Survey, we found a heterogeneous array of studies. A systematic review (34) examined the reliability and validity of the CTC Youth Survey and reported the following: Nine studies have been conducted in the United States (six of those for adaptation for other populations / cultural contexts) (11, 14, 35–41), three studies in Germany (23, 24, 42) and one each in South Africa (43), Colombia (44), Iran (45), Malaysia (46), and Trinidad and Tobago (47). These studies differ not only by country but also by the assessed domains of risk and protective factors, type of factor analysis used (34), and the composition and number of risk and protective factors in the assessed domains. Thurow et al. (34) conclude in their review that construct validity was generally adequate (although that implies that there were scales for which construct validity was not given) over diverse populations.

In previous research, some risk factors have similarly shown to be problematic regarding unidimensionality. Arthur et al. (11) calculated separate CFAs for each grade-sex combination for the 20 scales. The majority of the risk factors yielded satisfying results with regard to unidimensionality (11). However, early initiation of antisocial behaviour (IR3) also showed problems in its unidimensionality in that study (11). Accordingly, IR3 appears to be somewhat problematic regarding unidimensionality in both the U.S. version (11) and the German version. In both versions, this evidence was not as consistent (not all model fit indices, not all subgroups) as to warrant changing or removing this risk factor.

### 4.2 Validity

To distinguish the results, we considered associations to be present only if they showed at least a moderate strength of association. Nine out of 10 peer/individual risk factors were associated with violence, delinquency, and substance use indicating that peer/individual risk factors are good predictors of problem behaviour. In contrast, only one of the 10 peer/individual risk factors (rebelliousness) was associated with depressive symptomatology. Peer and individual risk factors have limited predictability on depressive symptomatology compared to problem behaviour. Most risk factors have a clear face validity linked to substance use or antisocial behaviour (directly assessing something related to these outcomes, e.g., peer drug use). However, rebelliousness (IR1) and sensation seeking (IR10) do not have a clear link to these outcomes.

Depending on the outcome, three to all of the five family risk factors predicted violence, substance use and delinquency. Depressive symptomatology was only predicted by family conflict (FR3). Again, most family risk factors are content-wise aiming at either substance use or antisocial behaviour. Only family conflict (FR3) and poor family management (FR2) have a different focus. Arthur et al. (11) assessed only correlations with the problem behaviours substance use and delinquency and report that poor family supervision, poor family discipline, family history of antisocial behaviour, and family attitudes favourable to antisocial behaviour were all moderately correlated with these.

Only delinquency was predicted by both school risk factors (academic failure and low school commitment); all other outcomes were predicted by only one school risk factor. Neither of these factors has a content-wise link to substance use or antisocial behaviour. In contrast, both school risk factors were moderately correlated with problem behaviours in previous research (11).

Community risk factors are good predictors of violence, delinquency, and substance use (three out of six; community disorganisation, CR2, perceived availability of drugs, CR4, perceived availability of handguns, CR5). Similarly, in previous research “perceived availability of drugs” showed one of the strongest associations with substance use and delinquency, whilst contrastingly, “community disorganisation” failed to show moderate correlations (11). Depressive symptomatology was only predicted by low neighbourhood attachment (CR1). Interestingly, a third pattern emerges in this domain: we find two risk factors not at least moderately predicting any outcome (transitions and mobility, CR3, and laws and norms favourable to antisocial behaviour, CR6). These were the only two predictors that were not at least moderately associated to one of the four outcomes. Thus, the usefulness of “transitions and mobility” and “laws and norms favourable to antisocial behaviour” as prediction factors is questionable.

No significant prediction could be made based on the following risk factors. There was no correlation between transitions and mobility (CR3) or laws and norms favourable to antisocial behaviour (CR6) and substance use. Additionally, there was no correlation between perceived risks of drug use (IR12) or perceived availability of handguns (CR5) and depressive symptomatology. Community risk factors seem to be less consistent predictors of problem behaviour and depressive symptomatology; possibly because the community context is furthest from the individual. Perceived risks of drug use (IR12), from a face validity point of view, seems to be a reasonable predictor of substance use but not depressive symptomatology. Previous research has shown that all risk factors have significant positive correlations with substance use and delinquency (11). Correlations were moderate to strong, with the exception of “community disorganisation” and “transitions and mobility” (11). “Transitions and mobility” therefore, appear to be problematic in both the German and the U.S. version.

The model could not be calculated for early initiation of antisocial behaviour (IR3) with violence and delinquency, as well as for early initiation of drug use (IR4) with substance use, due to the correlation approaching 1. A nearly perfect, though not very informative, correlation can be expected for current behaviour and early onset of the same behaviour.

In summary, the previous discussion points lead to the following overall conclusions: firstly, depressive symptomatology is not predicted as well as other problem areas. During the development phase of the CTC Youth Survey (11), risk factors were identified through literature reviews on adolescent drug use, delinquency, and violence, but not depressive symptomatology, which may explain why these are better predicted. Based on CTC practise, this has been overruled by communities using risk factor profiles to achieve better problem area outcomes (i.e., specific predictions of certain risk factors on only some problem areas are not taken into account). New problem areas, such as victim/survivor experience and indicators of wellbeing, have been identified over the years. These areas do not fit into the category of problem behaviour, and therefore lack strong theoretical underpinnings for the identification of relevant risk factors. The CTC Youth Survey initially included six outcomes: violence, delinquency, school drop-out, substance use, teenage pregnancy, and depressive symptomatology (11). However, previous CTC studies typically only report results on substance use and antisocial behaviour, rather than other CTC outcomes such as depressive symptomatology.

For instance, the European Monitoring Centre for Drugs and Drug Addiction (6) conducted a review on the reduction of substance use (incidence and prevalence), delinquency, and other problem behaviours. The review concluded that publications from the Community Youth Development Study, a randomised controlled community trial with 24 matched communities in the United States, mostly focused on substance use and delinquency/violence as outcomes (48). Similarly, the Pennsylvania Youth Survey (38) compared only substance use and delinquency outcomes between CTC and non-CTC communities.

The Alcohol Action in Rural Communities project (49), a cluster randomised controlled trial comprising 20 communities in Australia, focused solely on alcohol-related outcomes such as crime, traffic incidents, hospital inpatient admissions, risky consumption, and verbal abuse. Therefore, we recommend studying CTC outcomes such as depressive symptomatology, which have been part of the CTC Youth Survey from the beginning. Additionally, we suggest developing and including risk factors that predict these outcomes. Consideration should also be given to adding more outcomes related to wellbeing. We propose renaming the categorisation of outcomes, as becoming a victim or experiencing depressive symptoms should not be grouped as ‘problem behaviours’.

The second main conclusion is that correlations for problem behaviours are consistently higher for peer/individual risk factors than for other domains. This suggests that conceptualising the domains as a sequence ranging from proximal to distal may be valuable. As all outcomes are asking for individual behaviour/wellbeing/experience, a more direct (and thus stronger) criterion validity of peer/individual risk factors that ask for risks pertaining to the individual and not the community, family or school setting (etiologically, developmentally, temporally) is expectable (11). A previous study, in Germany, examined the transferability of risk factor cut-points (mean absolute deviation of the median) for substance use in terms of sensitivity, specificity, and criterion validity (42). Although our analyses used latent constructs instead of cut-points, it is worth noting that criterion validity was reported to be high, particularly for the peer/individual risk factors (42). Risk factors in the peer/individual domain showed higher correlations with problem behaviours than in the other domains (11). Additionally, community risk factors are not consistently predictive of problem behaviour and depressive symptomatology, possibly due to their distance from individual problem areas.

Early onset of a behaviour should not be modelled as a risk factor because past and present behaviours are nearly identical and previous behaviour is always a strong predictor of current behaviour, as shown e.g., in studies on the reasoned action approach. However, these scales showed concurrent criterion validity as risk factors for all other outcomes. It is debatable whether the onset of a problem behaviour should be interpreted as a risk factor. The onset of a behaviour is a necessary but not sufficient condition for developing problem behaviour. Therefore, we suggest removing these as risk factors from the CTC Youth Survey.

Finally, we found that every risk factor was correlated significantly with at least three problem areas. Therefore, one may conclude that concurrent criterion validity has been established for the German CTC Survey. However, when considering only moderate-to-strong relations, transitions and mobility (CR3) and laws and norms favourable to antisocial behaviour (CR6) did not sufficiently predict any outcome. Therefore, it may be considered to remove these risk factors from the German CTC survey.

### 4.3 Limitations

The CTC Youth Survey has undergone continuous adaptation in both its U.S. and German versions, including changes to both the scale and item levels. Some scales of the survey have been modified over time, with problematic factors being excluded and new ones being added (34). Therefore, when comparing the results of the original version by Arthur et al. (11) with the 2019 German version, some scales may differ. The current U.S. version (16) differs even more from the German version than the original final CTC Survey (11). Adaptations have also been made at the item level, which means that when comparing validity across studies, some items may diverge for reasons other than translation alone.

The data in this study were cross-sectional, therefore no causal relations between risk factors and problem areas can be assumed. It is worth noting that the majority of studies assessing the validity of the CTC Youth Survey have also been cross-sectional (17 out of 20; 34).

This study focuses solely on risk factors, as they may have a different prediction process than protective factors (“buffering”; 50). Additionally, we only assess the validity of single risk factors. Research on environmental risk factors in infancy suggests that the number of risk factors may be a better predictor of problem behaviour than singular risk factors (51).

The nested structure of the data (students within schools) was not considered, because the information on the schools was not available. According to the results of the multilevel logistic regression models in other research, some risk and protective factors showed significant variation across schools (11). It seems that there are significant risk/protection components at both levels (11).

Confounding variables cannot be excluded. The study only examined risk factors as single predictors and did not include additional sociodemographic variables. According to the CTC framework, these risk factors are universal (e.g., across grades).

The study is based on self-reported data. Whilst some items inquire about sensitive information and stigmatised behaviours, self-reported data may be more appropriate than parent-reported data when it comes to substance use.

### 4.4 Practical implications

The CTC Youth Survey aims to evaluate prevention needs (11), which is crucial for strategic prevention planning (11). It is important to ensure that the survey meets the validity requirements in the cultural context in which it is conducted. Communities are under increasing pressure to determine which prevention programmes should be implemented locally (6). To ensure coordinated and comprehensive prevention efforts, an effective needs assessment is required.

### 4.5 Future research

Future research should address the following topics: assessing the predictive criterion validity, as cross-sectional data only allows for the examination of correlative relationships and thus concurrent criterion validity. Additionally, the multilevel structure of the data should be taken into account. Furthermore, the validity of protective factors should be assessed, and their buffering effect should be modelled.

It remains unclear how CTC compares to other community-based interventions, or even which interventions are comparable. Gavine et al. (52) published a Cochrane protocol for “Universal community-based social development interventions for preventing community violence by young people,” but the editorial group later withdrew it. Therefore, we still lack a systematic review of this type of intervention.

## Data Availability

The datasets presented in this article are not readily available because data are available upon request due to ethical restrictions. Interested researchers may submit requests to the corresponding author for access to the data. All requests will be assessed by the Data Protection Officer of the University. Requests to access the datasets should be directed to Maren Reder, rederm@uni-hildesheim.de.
