# Supplementary material for: The German Communities That Care Youth Survey: dimensionality and validity of risk factors
Source: Front Public Health. 2024 Sep 30;12:1472347. doi: 10.3389/fpubh.2024.1472347 (PMC11471490; doi:10.3389/fpubh.2024.1472347)
Supplement: Supplementary file 1 [file Table_1.PDF]

Appendix A Additional File 1 — Unidimensionality supplement

Additional File 1 — Unidimensionality supplement

Scale		Item		λ
IR1	Rebelliousness	f27.5	I like to try out how far I can go.	.588
		f27.6	I don't stick to rules I don't like.	.640
		f27.8	I sometimes deliberately don't do what people tell me to do just to annoy them.	.690
IR3	Early initiation of antisocial behaviour	$f23.1.2_{cr}$	behaved in such a way that you were excluded from lessons by a conference?	.589
		$f23.2.2_{cr}$	deliberately broke something that doesn't belong to you?	.599
		$f23.3.2_{cr}$	participated in a violent or criminal youth gang?	.784
		$f23.4.2_{cr}$	stolen something from a shop?	.621
		$f23.5.2_{cr}$	been arrested by the police?	.705
		$f23.6.2_{cr}$	been involved in a fight?	.641
		$f23.7.2_{cr}$	assaulted someone in order to seriously injure him or her?	.620
		$f23.8.2_{cr}$	threatened someone to get money?	.664
		$f23.9.2_{cr}$	sold stolen things?	.871
		$f23.10.2_{cr}$	stolen something at school?	.560
		$f23.11.2_{cr}$	carried a weapon (e.g. a knife)?	.709
		$f23.12.2_{cr}$	sprayed graffiti on other people's property?	.651
		$f23.13.2_{cr}$	illegally downloaded music or films from the internet?	.484
		$f23.14.2_{cr}$	bullied someone at school or on the internet?	.594
IR4	Early initiation of drug use	$f37.1.2_{cr}$	drunk beer	.783
		$f37.2.2_{cr}$	drunk wine/champagne	.743
		$f37.3.2_{cr}$	drunk mixed drinks	.873
		$f37.4.2_{cr}$	drunk liquor	.873
		$f37.5.2_{cr}$	smoked cigarettes/tobacco (shisha, pipe snus)	.693
		$f37.7.2_{cr}$	hash/marijuana	.607
		$f37.8.2_{cr*}$	other illegal drugs (ecstasy, speed, LSD, cocaine, crystal or heroin)	–
		$f37.10.2_{cr*}$	prescription drugs without a prescription from a doctor (e.g. tranquillisers, stimulants or painkillers)	–
IR5	Attitudes favourable to drug use	f19.2	often drinks alcohol?	.768
		f19.3	does hash/marijuana?	.899

Scale		Item		λ
		f19.4	other illegal drugs (ecstasy, speed, LSD, cocaine, crystal, heroin)?	.867
		f19.1	smokes cigarettes?	.878
		f19.11	takes prescription drugs without a prescription from a doctor (e.g. tranquillisers, stimulants or painkillers)?	.440
IR6	Attitudes favourable to antisocial behaviour	f19.5	has a weapon with them (e.g. a knife)?	.683
		f19.6	steals something?	.806
		f19.7	starts a fight?	.789
		f19.8	attacks someone with the intention of physically harming him/her?	.814
		f19.9	skips school?	.612
IR7	Peer drug use	f20.1	smoked cigarettes.	.885
		f20.2	drunk alcohol.	.773
		f20.3	used hash/marijuana.	.904
		f20.4	taken other illegal drugs (ecstasy, speed, LSD, cocaine, crystal or heroin).	.853
IR8	Peer antisocial behaviour	f20.5	sold hasch/marihuana.	.821
		f20.6	sold other illegal drugs (ecstasy, speed, LSD, cocaine, crystal or heroin).	.841
		f20.7	skipped school frequently.	.614
		f20.8	carried a weapon (e.g., a knife)	.707
		f20.9	stolen something precious (e.g., a cell phone or a bike).	.789
		f20.10	arrested for a misdemeanor by police.	.881
		f20.11	left school without graduating.	.687
		f20.12	participated in a violent or criminal group (gang).	.785
IR9	Peer rewards for antisocial behaviour	f21.1	smoke cigarettes.	.849
		f21.2	drink alcohol	.766
		f21.3	use hash/marijuana.	.896
		f21.4	have a weapon with you (e.g. a knife).	.697
IR10	Sensation seeking	f29.1	I did what I enjoyed without considering the consequences.	.704
		f29.2	I did something dangerous because someone challenged me.	.798
		f29.3	I did crazy things, even if they were a bit dangerous.	.826

Additional File 1 — Unidimensionality supplement

Scale		Item		λ
IR12	Perceived risks of drug use	drugrisk1$_r$	smokes one or more packets of cigarettes a day?	.703
		drugrisk2$_r$	tried hash/marijuana once or twice?	.481
		drugrisk3$_r$	uses hash/marijuana regularly (once or twice a week)?	.688
		drugrisk4$_r$	drinks one or two glasses of alcohol (wine, beer) almost every day?	.650
FR1	Family history of antisocial behaviour	f44.1	used drugs?	.852
		f44.2	sold drugs?	.828
		f44.3	had a drinking problem?	.572
		f44.4	had a drug problem?	.978
FR2	Poor family management	f45.1$_r$	The rules in my family are clear.	.543
		f45.3$_r$	If I'm not at home, my parents know where I am.	.615
		f45.6$_r$	My parents want me to call if I come home late.	.482
		f45.7$_r$	My parents would find out if I take drugs or drink alcohol.	.700
		f45.8$_r$	My parents would find out if I skip school.	.632
		f45.9$_r$	In my family there are clear rules about alcohol and drugs.	.714
		f45.10$_r$	My parents ask me if I have done my homework.	.507
		f45.12$_r$	If I came home late, my parents would find out.	.546
FR3	Family conflict	f45.2	In my family we often shout at each other or call each other names.	.889
		f45.4	We always argue about the same things in our family.	.701
		f46.11	There is often a bad argument in our family.	.764
FR4	Parental attitudes favourable to drug use	f43.2	drink alcohol?	.699
		f43.3	smoke cigarettes?	.888
		f43.4	use hash/marijuana?	.788
FR5	Parental attitudes favourable to antisocial behaviour	f43.1	skip school?	.443
		f43.5	steal something?	.846
		f43.6	break things in your neighbourhood?	.716
		f43.7	get into a fight with someone?	.647
SR1	Academic failure	f15	How would you rate your overall school performance in the last year?	–
		f17.7	I often do worse at school than my classmates.	–

Scale		Item		λ
SR2	Low commitment to school	$f18.1_r$	At school I concentrate on the lessons.	.605
		$f18.2_r$	I try hard in my homework.	.548
		f18.3	I hate going to school.	.532
		$f18.4_r$	I find my homework useful and important.	.702
		$f18.5_r$	I like going to school very much.	.701
		$f16_c$	How many days have you skipped school in the last four weeks?	.487
		$f18.6_r$	I find the lessons very interesting most of the time.	.829
		$f18.7_r$	I find the things I learn at school important for my later life.	.679
CR1	Low neighborhood attachment	$f40.5_r$	I like my neighborhood.	.832
		f40.7	I'd like to get out of my neighborhood.	.811
		$f41.1_r$	If I had to move, I would miss the neighborhood I now live in.	.860
CR2	Community disorganisation	f42.1	There is a lot of unauthorised graffiti in my neighbourhood.	.713
		f42.2	In my neighbourhood there is a lot of rubbish on the street.	.626
		f42.3	In my neighbourhood there is a lot of crime, e.g. robberies and burglaries.	.798
		f42.4	There are people dealing drugs in my neighbourhood.	.794
		f42.5	There are often fights in my neighbourhood.	.933
		$f40.6_r$	I feel safe in my neighbourhood.	.451
CR3	Transitions and mobility	$f11_c$	How many times have you changed homes since kindergarten?	.932
		f12	Have you changed homes in the past year?	.668
		$f13_c$	How many times have you changed schools (including changing from elementary to middle or middle to high school) since kindergarten?	.597
		f14	Have you changed schools (including changing from elementary to middle or middle to high school) in the past year?	.689
CR4	Perceived availability of drugs	f39.1	marijuana?	.819
		f39.2	cocaine, LSD, or amphetamines?	.769
		f39.3	beer, wine, or hard liquor?	.870
		f39.4	cigarettes?	.908
		f39.7	prescription medicines without a prescription from a doctor?	.546

Scale		Item		λ
CR5	Perceived availability of handguns	f39.5	If you wanted to get a handgun, how easy would it be for you to get one?	–
CR6	Laws and norms favourable to anti-social behaviour	f40.1	If a young person or child in your neighbourhood breaks something, do the neighbours talk to him/her?	.742
		f40.2	If a young person or child in your neighbourhood treats other young people or children in a mean way: Do the neighbours speak to him/her?	.827
		f40.3	If there was a serious fight among the youths/children in your neighbourhood: Would the neighbours call the police?	.491
	Violence	f23.2.1	intentionally broken something that doesn't belong to you?	.633
		f23.3.1	joined a violent or criminal youth gang?	.847
		f23.6.1	been involved in a fight?	.721
		f23.7.1	attacked someone to seriously hurt him or her?	.669
		f23.8.1	threatened someone to get money?	.874
		f23.11.1	had a weapon with you (e.g. a knife)?	.705
	Delinquency	f23.4.1	stolen something from a store?	.731
		f23.5.1	been arrested by the police?	.805
		f23.9.1	sold stolen items?	.986
		f23.10.1	stolen something at school?	.679
		f23.12.1	sprayed graffiti on someone else's property?	.696
		f23.13.1	illegally downloaded music or movies from the internet?	.455
		f23.14.1	bullied someone at school or online?	.573
	Substance use	f37.1.1	beer	.659
		f37.2.1	wine/sparkling wine	.552
		f37.3.1	mixed drinks	.700
		f37.4.1	hard liquor	.672
		f37.5.1	cigarettes/tobacco (shisha, pipe, snus)	.937
		f37.7.1	hash/marijuana	.875
		f37.8.1	other illegal drugs (ecstasy, speed, LSD, cocaine, crystal, or heroin)	.787
		f37.10.1	prescription drugs without a prescription from a doctor (e.g. tranquillisers, stimulants, or painkillers)	.427
		f38	Please think back to the last four weeks. During this time, how often have you had 5 or more alcoholic drinks in one evening?	.672
	Depressive symptomatology	f27.9	Sometimes I think my life is worth nothing.	.949
		f27.10	Sometimes I think I'm good for nothing.	.925

Scale	Item		λ
	f27.11	I often think I'm a failure.	.954
	f27.12	For the past year, I've felt depressed or sad most days, although some days I've felt okay.	.786

*Note. c = categorised; r = recoded; * = excluded due to local misfit.*
