# Supplementary material for: The German Communities That Care Youth Survey: dimensionality and validity of risk factors
Source: Front Public Health. 2024 Sep 30;12:1472347. doi: 10.3389/fpubh.2024.1472347 (PMC11471490; doi:10.3389/fpubh.2024.1472347)
Supplement: Supplementary file 2 [file Table_2.PDF]

Additional File 2 — Concurrent criterion validity supplement.

Additional File 2 — Concurrent criterion validity supplement.

scale	n	free parameters	χ^2	df	p	CFI	RMSEA [90 % CI]	SRMR
IR1	1900	141	990.917	351	<.001	.989	.031 [.029, .033]	.097
IR3[1]	1900	209	785.827	307	<.001	.991	.029 [.026, .031]	.131
IR4[2]	1900	109	808.044	216	<.001	.985	.038 [.035, .041]	.107
IR5	1910	150	1911.888	407	<.001	.977	.044 [.042, .046]	.102
IR6	1910	150	1279.670	407	<.001	.985	.034 [.031, .036]	.093
IR7	1911	149	1704.899	379	<.001	.979	.043 [.041, .045]	.106
IR8	1911	174	1213.081	496	<.001	.987	.028 [.026, .029]	.088
IR9	1908	150	1533.985	378	<.001	.978	.040 [.038, .042]	.101
IR10	1900	144	972.062	351	<.001	.989	.031 [.028, .033]	.095
IR12	1902	146	1274.798	378	<.001	.985	.035 [.033, .037]	.096
FR1	1904	138	995.039	378	<.001	.989	.029 [.027, .032]	.097
FR2	1904	167	1451.465	495	<.001	.985	.032 [.030, .034]	.090
FR3	1904	141	939.055	351	<.001	.990	.030 [.027, .032]	.095
FR4	1905	141	1322.714	351	<.001	.984	.038 [.036, .040]	.105
FR5	1905	145	1195.375	379	<.001	.986	.034 [.031, .036]	.097
SR1	1904	139	912.174	324	<.001	.990	.031 [.029, .033]	.097
SR2	1911	173	1806.183	497	<.001	.980	.037 [.035, .039]	.092
CR1	1903	141	1002.257	351	<.001	.989	.031 [.029, .034]	.096
CR2	1903	156	1499.647	435	<.001	.982	.036 [.034, .038]	.095
CR3	1911	138	964.230	378	<.001	.990	.028 [.026, .031]	.098
CR4	1903	152	1394.758	405	<.001	.985	.036 [.034, .038]	.097
CR5[3]	1775	112	805.004	299	<.001	.990	.031 [.028, .033]	.108
CR6	1902	141	952.864	351	<.001	.990	.030 [.028, .032]	.096

Note. [1] Model calculation was only possible for substance use and depressive symptomatology due to non-convergence. [2] Model calculation was only possible for violence, delinquency, and depressive symptomatology due to non-convergence. [3] CR5 comprises only one item; this was dichotomised as hard/easy to allow calculation of concurrent criterion validity. Due to no or very few cases in the substance use categories indicating frequent use, model calculation was only possible when those substance use categories were collapsed to > 6 time in the last 4 weeks.
